# *A*‑Site and Epitaxial Strain Effect on the Properties of *A*Mn_3_Sb_5_ (*A* = K, Cs, and Rb) Kagome Lattices

**DOI:** 10.1021/acsomega.5c10090

**Published:** 2026-01-23

**Authors:** Andrés Camilo García Castro, Wilfredo Ibarra Hernández

**Affiliations:** † School of Physics, 28014 Universidad Industrial de Santander, Bucaramanga Santander SAN-680002, Colombia; ‡ Facultad de Ingeniería, 3972 Benemérita Universidad Autónoma de Puebla, Apartado Postal J-39, Puebla, Puebla 72570, México

## Abstract

Kagome lattices are an exciting family of compounds where frustrated magnetism, superconductivity, charge density wave (CDW) orders, topological features, large anomalous Hall conductivity, flat bands, and van Hove singularities collide. With this in mind, we theoretically studied the Mn-based Kagome *A*Mn_3_Sb_5_ (*A* = K, Rb, and Cs) lattices and explored the *A*-site’s influence, as well as the epitaxial *xy*-strain effect on the structural, magnetic, and electronic properties. Through theoretical analysis, coupled with first-principles density functional theory calculations, we demonstrate that the entire family is dynamically stable, locking the ferromagnetic order as the ground-state configuration in the primitive cell and preventing charge-density wave condensation, as observed in the *A*V_3_Sb_5_ family. In these compounds, our results suggest an out-of-plane ferromagnetic response that, in turn, breaks the T -symmetry, allowing a nonzero Berry curvature. The latter leads to a symmetry-allowed anomalous Hall conductivity (σ_*αβ*_) as large as σ_*xy*_ = 402 S·cm^–1^ in the CsMn_3_Sb_5_ and σ_*xy*_ = 442 S·cm^–1^ under +3% in-plane epitaxial strain.

## Graphical abstract

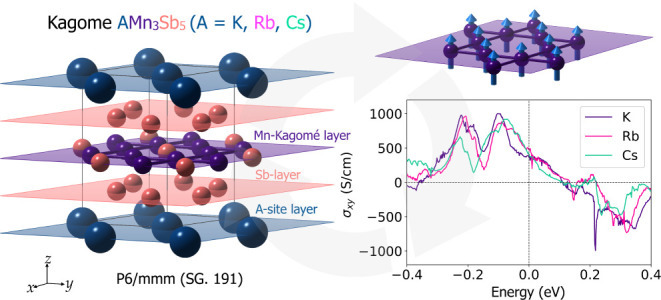

## Introduction

Novel magnetically active quantum materials in which topological features are present are at the center of attention in the condensed matter field. Among these materials, Kagome lattices^1–3^ stand out due to their exciting electronic phenomena that include charge-density waves,^4,5^ Van-Hove singularities^6–9^ magnetic frustration leading to noncollinear antiferromagnetic orderings,^10^ electronic flat bands,^11^ superconductivity,^12^ among others.^13^ The latter phenomena are observed in several Kagome-like materials, for example FeSn,^14^ Co_3_Sn_2_S_2_,^15^ ScV_6_Sn_6_,^16^ and Mn_3_ *A*N (*A* = Ni, Ga, Pt, Pd, and Sn) and V_3_AuN antiperovskites.^17–22^

Particularly, the Kagome lattice offers symmetry features that involve triangularly coordinated magnetically active cations, giving rise to several properties, such as frustrated magnetism and topology. In recent years, the *A*V_3_Sb_5_ (*A* = K, Rb, and Cs) family has drawn tremendous attention because it materializes these aforementioned advantages.^23–27^ Other material prototypes within the stoichiometric and symmetry contemplate compounds as *A*Ti_3_Bi_5_ ^28–30^ where superconductivity and charge-density wave states have also been suggested in the absence of long-range magnetic order due to the substitution of the metallic Kagome center by a nonmagnetically active Ti site. Unfortunately, the absence of ferromagnetic behavior and magnetically active long-range ordering has been recently confirmed in the *A*V_3_Sb_5_ family, supporting weak electronic correlations.^31–33^ In consequence, the study of the magnetically active Kagome lattice, with a tangible presence of long-range interactions, opens the possibility for studying the entanglement between the quantum magnetism and topological features in these lattices. In this arena, *R*Mn_6_Sn_6_ family of crystals, with *R* rare-earth elements such as Y, Gd, Tb, and Ho^34^ have appeared to provide a stable playground for the exploration of magnetically active Kagome crystals. Consequently, in this type of compound, ferromagnetic and noncollinear antiferromagnetic states can be expected to stabilize, leading to symmetry-allowed topological features related to Dirac and Weyl Fermions in the vicinity of the Fermi level. The latter results in nonvanishing Berry curvature inducing observables such as anomalous Hall conductivity (AHC) and nonlinear Hall effects.^35–39^ Importantly, the electrical manipulation of the Berry curvature-induced anomalous Hall effect at room temperature offers unimaginable capabilities in future spintronic devices.^15–42^ Therefore, ferromagnetic Kagome metals, with access to reversible magnetization, can be used as an external parameter to control and switch the AHC response. Furthermore, discovering novel Kagome materials with out-of-plane magnetization is the key to exploring massive Dirac Fermions in the Haldane model.^43^ On these grounds, the search for novel candidates within the quasi-2D ferromagnetic Kagome materials, belonging to the *AM*_3_Sb_5_ (with *A* = K, Rb, Cs, *M* = V, Cr, Fe, and Mn) stoichiometry, could lead to the discovery and better understanding of quantum phases driven by nontrivial electronic, topological, magnetic, and superconducting properties. Additionally, these electronic and magnetic properties in these compounds are shown to be highly sensitive to external constraints, such as epitaxial strain, and thus such external features can be explored in order to gain better control of the properties shown.^20,44–46^

In this work, we employed first-principles density functional theory (DFT) calculations to delve into the *A*-site and epitaxial *xy*-strain in the structural, vibrational, magnetic, and topological features of the Kagome metal family *A*Mn_3_Sb_5_ with *A* = K, Rb, and Cs.^47^ Our results showed that even though the noncollinear antiferromagnetic orderings are allowed by symmetry due to the triangular Mn coordination, the *A*Mn_3_Sb_5_ exhibits a stable ferromagnetic ground state in the unit cell, with the magnetic easy axis oriented along the *z*-axis. As such, the time-reversal symmetry breaking unlocks the existence of massive topological Dirac nodes in this centrosymmetric crystal, leading to the large appearance of the Berry curvature and associated anomalous Hall conductivity. Therefore, our research provides theoretical insights into the physics beneath the *A*Mn_3_Sb_3_ (*A* = K, Rb, and Cs) family, standing as a novel and promising Kagome compounds, in which the absence of rare-earth elements and simple stoichiometric formulas, a strong ferromagnetic response with an out-of-plane easy-axis, and tangible large AHC among the Kagome-like symmetries make them attractive in the field. Furthermore, our results provide valuable information for experimentalists in synthesizing and confirming the predicted properties of the Kagome *A*Mn_3_Sb_5_ family. Thus, in the first section, we provide details of the computational methods and theoretical approaches used in this study that involve symmetry considerations and first-principles density functional calculations. This is followed by a section that condenses the main results focused on the *A*-sites and epitaxial *xy*-strain effects on the structural, magnetic, and electronic properties. Finally, we draw our conclusions and discuss perspectives on the novel *A*Mn_3_Sb_5_ (*A* = K, Rb, and Cs) family of materials.

## Theoretical and Computational Approaches

We performed a theoretical analysis based on first-principles calculations within the Density-functional theory approach.^48,49^ We used the projected-augmented wave (PAW)^50^ method as implemented in the VASP code (version 5.4.4).^51,52^ The valences’ electronic configurations considered in the PAW pseudopotentials are as follows: K: (3*p* ^6^4*s* ^1^, version 17Jan2003), Rb: (4*p* ^6^4*s* ^2^5*s* ^1^, version 06Sep2000), Cs: (5*p* ^6^5*s* ^2^6*s* ^1^, version 08Apr2002), Mn: (3*p* ^6^3*d* ^5^4*s* ^2^, version 02Aug2007), and Sb: (5*s* ^2^5*p* ^3^, version 06Sep2000). The exchange-correlation functional was computed using the generalized-gradient approximation as parametrized by Perdew–Burke–Ernzerhof for solids (GGA-PBEsol).^53^ The reciprocal space was sampled using a Γ-centered Monkhorst–Pack *k*-mesh^54^ of size (11 × 11 × 9), and a kinetic energy cutoff of 600 eV was used for the plane wave basis set. These values resulted in the convergence of residual forces and total energy to better than 0.001 eV·Å^–1^ and 0.1 meV. Spin–orbit coupling (SOC) was included to properly consider noncollinear magnetic configurations.^55^ Phonon calculations were performed within the finite differences approach^56,57^ and postprocessed using the PHONOPY code.^58^ To compute the anomalous Hall conductivity and Berry curvature, we utilized the Wannier functions methodology, for which the wannierization was performed using the WANNIER90 code^59,60^ and postprocessed with the WANNIERBERRI package.^61^ In this step, a 320 × 320 × 320 *k*-mesh was used in the Kubo’s relationship defined as^62,63^

1 $$\sigma_{\alpha \beta }=-\frac{e^{2}}{{\left(2\pi \right)}^{3}\hbar }\overset{occ}{\underset{n}{\sum }}\int_{BZ}f_{n}\left(k\right)\Omega_{n,\alpha \beta }\left(k\right)d^{3}k$$

in which the Berry curvature is defined as $\Omega_{\alpha \beta }\left(k\right)=\overset{occ}{\underset{n}{\sum }}f_{n}\left(k\right)\Omega_{n,\alpha \beta }\left(k\right)$ and calculated in the *αβ*-plane and results from the consecutive summation over all the occupied *n*-bands where *f*_*n*_ **(k)** represents the Fermi distribution. In these calculations, the Ω_*n,αβ*_(**k**) Berry curvature is estimated such as

2 $$\Omega_{n,\alpha \beta }\left(k\right)=-2i\hbar^{2}\underset{m\neq n}{\sum }\frac{\left\langle \psi_{n,k}\left|v_{\alpha }\right|\psi_{m,k}\right\rangle \left\langle \psi_{m,k}\left|v_{\beta }\right|\psi_{n,k}\right\rangle }{{[E_{m}(k)-E_{n}(k)]}^{2}}$$

In the latter equation, ψ_*n*,**k**_ and *v*_α_, *v*_β_ are the Bloch functions and the velocity operators, respectively. For the wannierization process, orbitals *s* and *p* were considered for the *A*-sites (K, Rb, and Cs) and the Sb atoms, while *s*, *p*, and *d* were considered for the Mn sites. Finally, the crystal structures were visualized using the VESTA software,^64^ and the data were postprocessed using the PYPROCAR^65^ software.

## Results and Discussion

*A*Mn_3_Sb_5_ layered Kagome family crystallizes in the hexagonal *P*6/*mmm* No. 191 space group, SG. In this structure, the Mn sites form the Kagome lattice in the middle of the *z*-axis (0,0,1/2). Here, the Sb sites are embedded in the central Kagome hexagon (*z* = 1/2) as well as in graphite-like Sb layers in positions for the *z*-plane *z* = 1/4 and *z* = 3/4 as displayed in Figure 1a. Finally, the *A*-sites (*A* = K, Rb, and Cs) complete the crystal and hold the (0, 0, 0) atomic positions. In all the cases, KMn_3_Sb_5_, RbMn_3_Sb_5_, and CsMn_3_Sb_5_, we performed a complete electronic and atomic relaxation, finding lattice parameters of *a* = 5.337 Å, 5.346 Å, and 5.354 Å, respectively (also included in Figure 2). As observed, the *xy*-plane dimensions remain almost constant, suggesting the dominance of the Mn-Kagome lattice in the in-plane correlations. In contrast, the relaxed *c* lattice parameters are *c* = 9.028 Å, 9.238 Å, and 9.508 Å for the KMn_3_Sb_5_, RbMn_3_Sb_5_, and CsMn_3_Sb_5_, respectively. This shows a tangible elongation as a function of the ionic *A*-site radius size as expected when going from K (*r*_K_ = 152 pm) to Cs (*r*_Cs_ = 181 pm).^66^ In Figure 1c, we show the computed X-ray diffraction, XRD, pattern in the θ–2θ configuration. As expected from the relaxed lattice parameters and the symmetry group in the *A*Mn_3_Sb_5_ family, it is observed that a shift to higher θ values is observed for KMn_3_Sb_5_ for the smaller *c* lattice parameters. Following the magnetic analysis, with a magnetic moment arising from the magnetically active Mn sites, we define all the symmetry-allowed noncollinear magnetic states that can be associated with a **q** propagation vector of (0, 0, 0). We find that, similar to the case of antiperovskites,^22^ four *xy* in-plane noncollinear chiral antiferromagnetic (AFM) orderings are allowed, as shown in Figure 1b. To these orderings are added the *z*-axis out-of-plane and *xy*-plane-oriented ferromagnetic (FM) state. These noncollinear antiferromagnetic orderings are identified as Γ_4*g*_, Γ_5*g*_, Γ_6*g,x*_, and Γ_*6g,y*_ (see Figure 1b), similarly, as in the case of antiperovskite Mn_3_ *A*N compounds.^67^ Interestingly, these AFM orderings hold a magnetic vector chirality, defined as $\kappa =\frac{2}{3\sqrt{3}}\underset{i,j}{\sum }\left(S_{i}\times S_{j}\right)$ for the Kagome lattice, where the *i* and *j* indices run over the magnetic moments in the unit cell. In an attempt to explore the noncollinear magnetic lowest-energy orderings, we computed the total energy of each compound under the allowed orderings. We found that, in all cases, the lowest-energy configuration belongs to the ferromagnetic state when compared to the chiral in-plane antiferromagnetic orderings displayed in Figure 1b. As such, for KMn_3_Sb_5_ the energy differences, taken as Δ*E* = *E*_FM001_ – *E*_noncol‑AFM_, are between −543 and −555 meV·f.u^.–1^. Similarly, for RbMn_3_Sb_5_ and CsMn_3_Sb_5_ Δ*E* values are between −548 and −561 meV·f.u^.–1^ and between −558 and −573 meV·f.u^.–1^, respectively.

**Figure 1:**
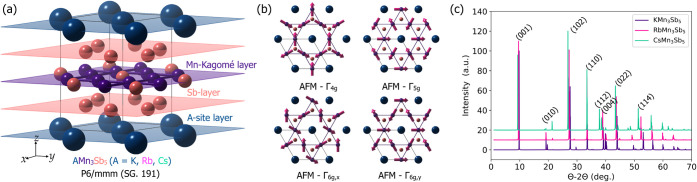
(a) Crystallographic *A*Mn_3_Sb_5_ (*A* = K, Rb, and Cs) and *P*6/*mmm* (SG. 191) hexagonal Kagome structure obtained for the ferromagnetic ordering, (001) FM. In this structure, the *A*, Mn, and Sb sites are denoted in navy, violet, and pink colors, respectively. In (b), we display, in a (2 × 2 × 1) cell, the chiral noncollinear antiferromagnetic orderings allowed in the *A*Mn_3_Sb_5_ magnetically frustrated Kagome structure. For those orderings, the Γ_4*g*_ and Γ_5*g*_ states show a +1 magnetic chirality, whereas the Γ_6*g*,*x*_ and Γ_6*g*,*y*_ AFM orders inherit a −1 magnetic chirality. (c) Computed X-ray diffraction patterns, considering Cu:Kα of 1.5406 Å, for the KMn_3_Sb_5_, RbMn_3_Sb_5_, and CsMn_3_Sb_5_ compounds, respectively.

**Figure 2:**
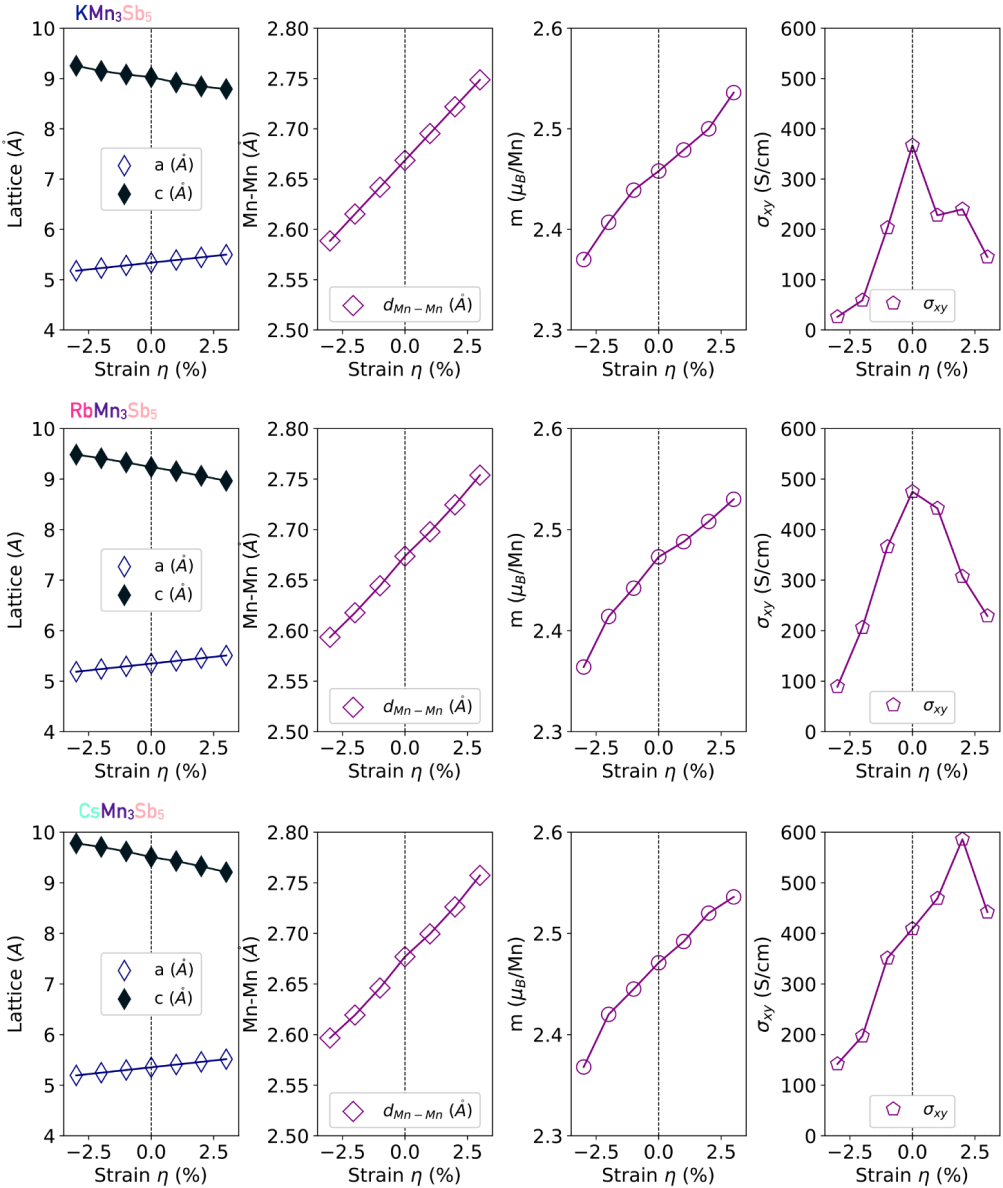
Lattice parameters (*a* = *b*, and *c*), manganese magnetic moments, *m*_*z*_, Mn–Mn distance, *d*_Mn‑–Mn_, and σ_*αβ*_ anomalous Hall conductivity σ_*xy*_ component. All computed as a function of the *xy* in-plane epitaxial strain for the different *A*-sites in the *A*Mn_3_Sb_5_ Kagome family when considering the lower energy out-of-plane ferromagnetic ordering in the **q**-vector of the (0,0,0) propagation vector.

Although more complex orderings, such as spin-spiral states, could appear at larger propagation distances in real space, the energy differences between the in-plane noncollinear AFM and the out-of-plane FM suggest otherwise. The FM state lies much lower in energy. Therefore, despite the symmetry-allowed linear combinations of the in-plane noncollinear antiferromagnetic orderings, the prediction of the long-range spiral states is unlikely to be captured in the theoretical framework of our work. Nonetheless, further experimentally focused works could shed light on deeper details of the magnetic structure of the *A*Mn_3_Sb_5_ family of compounds.

Our results suggest that in this 2D-like stacking, the breaking of Mn–Mn bonding along the out-of-plane direction affects the magnetic frustration. The latter symmetry breaking could lower the potential energy for the magnetocrystalline anisotropy, resulting in the breaking of magnetic frustration and the stabilization of ferromagnetic exchange interactions. This is in direct contrast to the 3D arrangement of the Kagome lattices, in which, for example, the frustration is preserved as in magnetically active antiperovskite compounds.^20,22^

To gain a better understanding of the magnetic easy axis, we investigated the preferred direction in which the magnetic moments tend to align with respect to the Mn Kagome plane. Specifically, we examined whether the moments lie in the *xy*-plane (within the Kagome plane) or if those moments are oriented perpendicular to it (along the *z*-axis). Based on the total energy per formula unit calculated, Δ*E*_1_ in Table 1, our results reveal that in all the cases, for *A* = K, Rb, and Cs without strain, the total magnetization is aligned toward the *z*-axis and out-of-plane of the Kagome lattice. Therefore, the following findings and discussion will be focused on the (001) ferromagnetic low-energy state of the Kagome *A*Mn_3_Sb_5_ for **q** = (0,0,0). We observe that, in all cases in the absence of epitaxial strain, the magnetic moment is in an approximated value of 2.5 μ_B_ · Mn^–1^ (see Figure 2) which is in the same range with the reported value in the Mn-based Kagome TbMn_6_Sn_6_ of 2.4 μ_B_ · Mn^–1^ measured by the neutron diffraction technique.^68^

**Table 1:** Energy Difference Δ*E*_1_ Is Equal to *E*_FM001_ – *E*_FM100_ Estimated in the Unit Cell, Meanwhile the Δ*E*_2_ Values Are Considered as *E*_AFM001_ – *E*_FM001_ Where the AFM Is Obtained for a **q**-Vector of (0,0,1/2)

Strain (%)	Δ*E*_1_ (meV·f.u^.–1^)	Δ*E*_2_ (meV·f.u^.–1^)
KMn_3_Sb_5_		
–3.0	+0.236	–2.845
0.0	–1.254	–3.773
+3.0	–1.373	–2.297
RbMn_3_Sb_5_		
–3.0	+0.127	–1.351
0.0	–1.310	–1.799
+3.0	–1.532	–1.046
CsMn_3_Sb_5_		
–3.0	+0.083	+0.869
0.0	–1.330	+0.170
+3.0	–1.786	+1.882

In the strained crystal cases, which can be achieved by thin-film growth, for example, in the hybrid perovskite cases,^69^ we applied the *xy*-strain as follows:

3 $$\eta =\frac{a-a_{0}}{a_{0}}\times 100\%$$

In which the *a*_0_ value refers to the fully relaxed *a* lattice parameter in each of the *A*Mn_3_Sb_5_ crystals. As such, the *a* lattice parameters are fixed, see Figure 2, meanwhile the *z* lattice parameter and the internal atomic positions are allowed to fully relax. The fully relaxed *c* lattice parameter, included in Figure 2, showed that, as expected from the Poisson ratio, the out-of-plane lattice is expanded (compressed) at negative (positive) strain η values. This epitaxial strain then alters the Mn–Mn distance, as shown in Figure 2, and then modifies the magnetic exchange interaction, which in turn changes the magnetic moment at the Mn sites. Thus, the values vary from 2.370 μ_B_ · Mn^–1^ at −3% to 2.536 μ_B_ · Mn^–1^ at +3% of epitaxial strain.

Motivated by several reports in which magnetically active Kagome lattices displayed *xy*-planar AFM coupling despite the expected weak out-of-plane magnetic interaction,^70^ we explored the possible AFM coupling between Kagome lattices for a **q** = (0,0,1/2) propagation vector. To this end, we computed the total energy per formula unit and compared it with the full ferromagnetic interaction, such as Δ*E*_2_ = *E*_AFM_ – *E*_FM_, included in Table 1 and extracted for all of the compounds and considering the *xy*-plane epitaxial strain. We observed that, in the KMn_3_Sb_5_ and RbMn_3_Sb_5_ cases, at larger propagation vectors, as in **q** = (0,0,1/2), the out-of-plane *A*-type AFM is slightly lower in energy. In the CsMn_3_Sb_5_ crystal, the result suggests stable out-of-plane ferromagnetic ordering. Interestingly, the energy value differences follow the *c* lattice parameter trend: as smaller is the *c* lattice parameter, as in KMn_3_Sb_5_ with *c* = 9.028 Å, the stronger the out-of-plane AFM exchange interaction. Thus, for large *c* values as in the CsMn_3_Sb_5_ (*c* = 9.508 Å), the FM exchange interaction is stronger. To better understand the magnetic behavior in the interlayer interaction and by using an exchange Hamiltonian in the form *H* = ∑ *J*_*ij*_ *S*_*i*_ · *S*_j_, where the *S*_*i,j*_ runs over the magnetic moments per Mn site, we obtained the exchange constants of computing the interlayer exchange constants of *J*_interlayer_ = −0.21 meV, −0.10 meV, and +0.01 meV for the KMn_3_Sb_5_, RbMn_3_Sb_5_, and CsMn_3_Sb_5_, respectively. The latter values suggest a low-temperature AFM interlayer coupling and, therefore, an FM response expected at higher temperature regimes. Therefore, the selection of *A* sites in this family of Kagome compounds can be used as a mechanism to tune the magnetic interaction by modification of the *c* lattice parameter and, with it, the out-of-plane magnetic interactions.

One of the key features in the description of novel systems is the confirmation of the dynamical stability, as this type of compounds tends to show phonon-driven charge-density wave states, as in the *A*V_3_Sb_5_ materials.^24,26,27^ Therefore, we evaluated the dynamical stability of the ground-state noncollinear (001) FM order in all of the *A*Mn_3_Sb_5_ compounds. In Figure 3 we present the obtained phononic dispersion of the fully relaxed structures of KMn_3_Sb_5_, RbMn_3_Sb_5_, and CsMn_3_Sb_5_ Kagome crystals. In these cases, we projected the atomic contribution to each phonon branch displayed along the high-symmetry path of the Brillouin zone for the *P*6/*mmm* space group. Here, the K, Rb, and Cs contributions are shown in navy, red, and green, respectively, whereas the Mn and Sb sites are shown in violet and pink for notation. At first glance, we obtained a completely stable phononic spectrum, confirming the dynamical stability of these Kagome lattices at low temperature and theoretically neglecting the existence of a displacive group-to-subgroup phase transition. As such, the formation of charge-density waves driven by the phonon modes is not expected in the Mn-based Kagome lattices presented here, in direct contrast to the *A*V_3_Sb_5_ family of Kagome compounds. Moreover, analyzing the atomically projected states, it can be noted that the *A*-site vibrational states are strongly localized between 50 and 70 cm^–1^ approximately, suggesting localized phononic states with small contribution to the thermal activity of these compounds. On the other hand, the Sb states dominate the frequency range from 0 cm^–1^ to approximately 150 cm^–1^ in all the cases, followed by the Mn-driven vibrational states above 150 cm^–1^ up to a value of 250 cm^–1^. Thus, our results suggest that the *A*-sites help to stabilize the crystal but play a minor role in the thermal activity. In addition, all the phononic landscapes in all of the compounds are similar and are dominated by the Sb and Mn sites.

**Figure 3:**
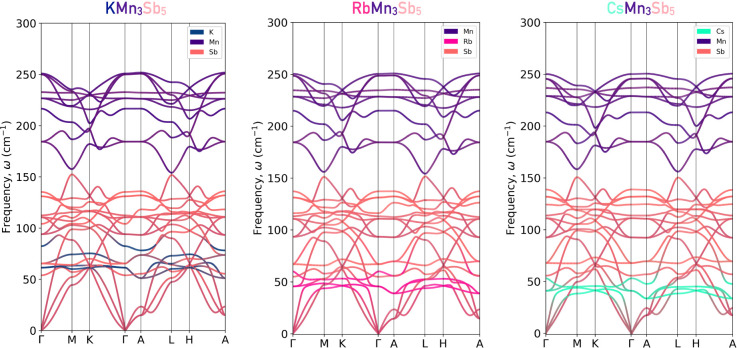
Full phonon-dispersion curves including the atomically projected phononic states obtained at the KMn_3_Sb_5_, RbMn_3_Sb_5_, and CsMn_3_Sb_5_ Kagome compounds. Here, it can be noticed that the Mn-Kagome sites are predominantly localized at high-frequency values, whereas the *A*-sites are mostly localized between 40 and 60 cm^–1^ for all the cases. Finally, the Sb contributions are spread between 0 and approximately 150 cm^–1^.

Finally, the *A*-site atom’s contribution to the lattice dynamics is strongly localized around 60 cm^–1^. To prove that no CDW or other type of phonon-driven displacive phase transitions can be induced by the strain, we have also computed the full phonon dispersion curves, in each case, as a function of the epitaxial *xy*-plane strain, as shown in Figure S1 in Supporting Information.^71^ Moreover, the expansion (compression) strain values generate a softening (hardening) of the vibrational modes in each Kagome *A*Mn_3_Sb_5_ crystal.

Regarding the electronic structure, in Figure 4 we present the computed electronic band structure of CsMn_3_Sb_5_ for discussion purposes. The electronic band structures of KMn_3_Sb_5_ and RbMn_3_Sb_5_ are closely similar and therefore are not included. However, they are included in the spin-polarized electronic band structure in Figure S3 in the Supporting Information,^71^ discussed later. In these calculations, the noncollinear ordering belongs to the (001) ferromagnetic arrangement with magnetic moments pointing toward the *z*-axis. Moreover, the effect of the spin–orbit coupling, SOC, is taken into account in all calculations. In this case, we showed selective Mn:3*d* orbital-projected bands where the in-plane: $d_{xy}+d_{x^{2}-y^{2}}$ , and out-of-plane: *d*_*xz*_+*d*_*yz*_ and $d_{z^{2}}$ are shown. As can be noted, for the $d_{xy}+d_{x^{2}-y^{2}}$ , *d*_*xz*_+*d*_*yz*_, and $d_{z^{2}}$ projected orbitals, the expected Kagome-like band profile is observed in the vicinity of the Fermi energy, here at 0 eV for notation. The $d_{xy}+d_{x^{2}-y^{2}}$ orbitals show an occupied state characteristic of the Kagome lattices with a gapped Dirac point at the *K* and *H* high-symmetry points. These gapped points are located at an energy of −1.5 eV at the Fermi level. Additionally, the quasi-flat bands are observed at an energy close to −1.0 eV. The latter gapped Dirac nodes originate from the degeneracy lifting of the protected points and are explained in terms of the spin–orbit effect and the magnetically active lattice that induced an additional symmetry breaking.^43^

**Figure 4:**
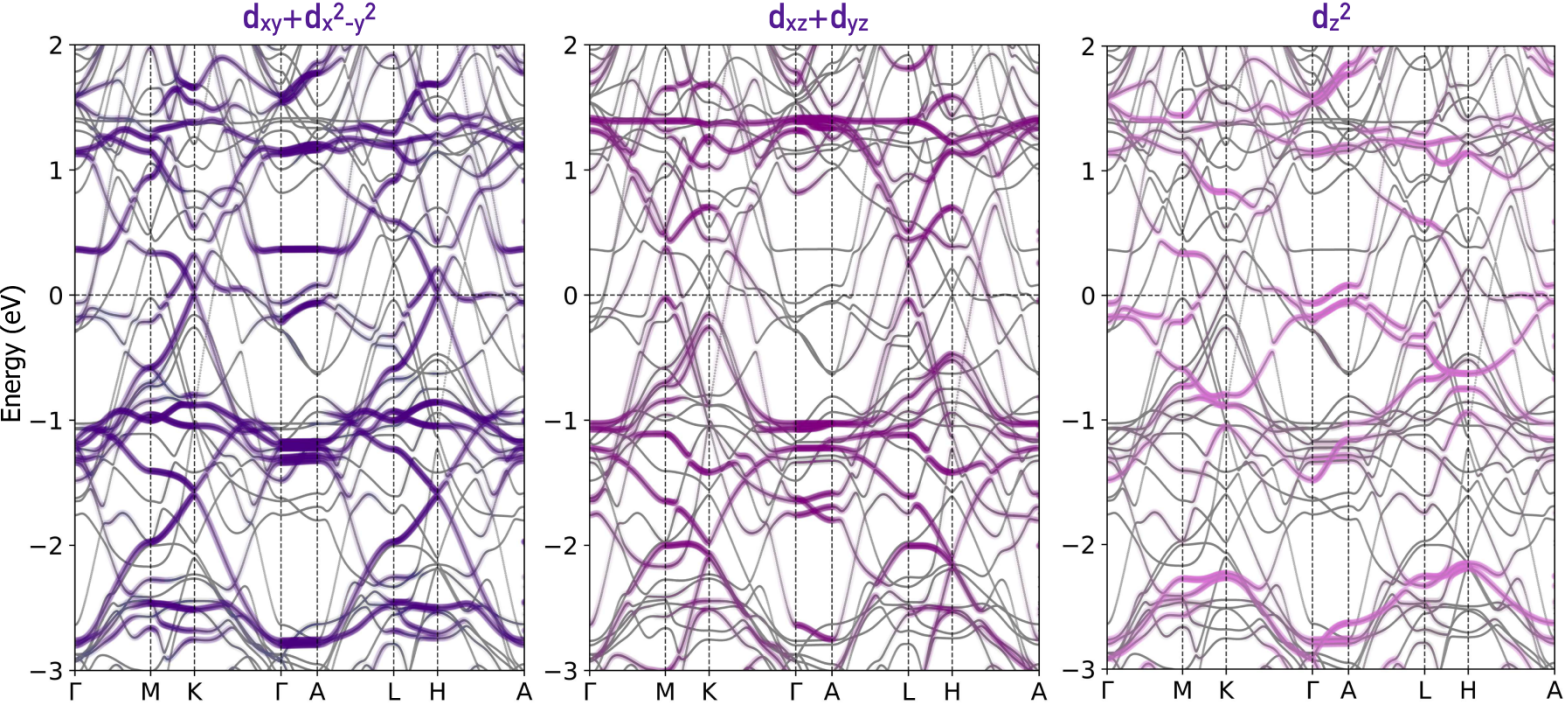
CsMn_3_Sb_5_ Mn:3*d* orbital-projected electronic band structure computed in the (001) noncollinear ferromagnetic configuration. These calculations consider the spin–orbit coupling, SOC, effect. Here, the in-plane: $d_{xy}+d_{x^{2}-y^{2}}$ , and out-of-plane: *d*_*xz*_+*d*_*yz*_ and $d_{z^{2}}$ orbitals contributions are shown separately. As expected, for the $d_{xy}+d_{x^{2}-y^{2}}$ and *d*_*xz*_+*d*_*yz*_ projected orbitals, the expected Kagome-like band profile is observed.

Once the spin-projected electronic structure is analyzed, in Figure S3 in Supporting Information,^71^ we noticed the emergence of several Weyl nodes in the vicinity of these *K* and *H* high-symmetry points, away from the Fermi level, and moved from the high-symmetry *k*-path considered in Figure 3b.^43,72,73^ As expected, the symmetry-protected nodes appear along the high-symmetry directions of the crystal’s Brillouin zone, as anticipated from the Kagome lattice symmetry.^15,74^ Some of these nodes are located along the Γ–*M*–*K k*-path at energies close to 150 meV above the Fermi level reference. Moreover, we observed symmetry-protected nodes located at the *L*–*H*–*A* path belonging to the (0, 0, 1/2) *k*-plane.

In the case of the strained crystals, shown in Figure S2 in Supporting Information^71^ for CsMn_3_Sb_5_, the electronic structure is qualitatively the same, as this applied strain, considered as an external perturbation, does not alter the crystallographic Kagome space group and associated symmetry operations. Nonetheless, the in-plane strain shows an effect on the tuning of the electronic structure by displacing to lower energy values the states when compression is applied in the *xy*-plane. Then the gapped Dirac points lie approximately −10 meV below the Fermi level at −3% of strain. On the contrary, for positive values, +3%, the gapped points are almost +20 meV above the Fermi energy. The opposite effect is observed for the electronic states along the high-symmetry axis and along the Γ – *A* path. Therefore, the epitaxial strain can be used to fine-tune the electronic states near the Fermi energy.

In this case, the presence of the gapped Dirac and Weyl points, added to the breaking of the time-reversal symmetry, leads to a nonzero Berry curvature. The latter is then driving the symmetry-allowed anomalous Hall conductivity, AHC, for the ferromagnetic ordering in the Kagome *A*Mn_3_Sb_5_ magnetic symmetry group, MSG, *P6/mm*′*m*′ (MSG. 191.240). Thus, the symmetry-allowed AHC tensor (σ_*αβ*_) is extracted and is presented in eq 4, in which most of the tensor elements are forced to vanish due to symmetry considerations. The nonzero components are the σ_*xy*_ and σ_*yx*_ and they follow the relationship σ_*yx*_ = – σ_*xy*_.^75,76^

4 $$\sigma_{6/mm\prime m\prime }=\left(\begin{array}{ccc} 0 & \sigma_{xy} & 0\\ -\sigma_{xy} & 0 & 0\\ 0 & 0 & 0 \end{array}\right)$$

The σ_*xy*_ conductivity component, considered as a function of energy near the Fermi level, is obtained by following Kubo’s relationship^62,63^ as commented on in the computational and theoretical details section. The latter is presented for all the compounds and at strain values from −3% to +3% to better capture the evolution with the epitaxial strain as an external perturbation. In Figure 5, we present the computed σ_*xy*_ curves, as a function of energy near the Fermi level, for KMn_3_Sb_5_, RbMn_3_Sb_5_, and CsMn_3_Sb_5_. At the Fermi level and in the unstrained crystals, the obtained values are σ_*xy*_ = 367 S·cm^–1^, 475 S·cm^–1^, and 442 S·cm^–1^ for the KMn_3_Sb_5_, RbMn_3_Sb_5_, and CsMn_3_Sb_5_ compounds, respectively, values also shown in Figure 2. In all of the compounds, there is a presence of strong peaks of AHC values reaching up to 1000 S·cm^–1^ at energies close to −100 and −200 meV, as appreciated from Figure 5. These peaks are displaced to lower energy values at compression strain values, with respect to the Fermi level (shown by the gray arrows in Figure 5), meanwhile they are moved closer to the *E*_*F*_ at expansion values added to broadening at positive η. Importantly, the computed values are in the range of similar Kagome crystals with reported large anomalous Hall conductivity values, as, for example, σ_*xy*_ = 223 S·cm^–1^ in GdMn_6_Sn_6_,^77^ σ_*xy*_ = −400 S·cm^–1^ in Fe_3_Sn_2_,^78^ and σ_*xy*_ = 1130 S·cm^–1^ in Co_3_Sn_2_S_2_.^15^ In the strained lattices, we observed that at compression strain values, +3%, the AHC drastically diminishes the σ_*xy*_ components up to 26, 89, and 142 S·cm^–1^ for the KMn_3_Sb_5_, RbMn_3_Sb_5_, and CsMn_3_Sb_5_ compounds, respectively, see Figure 2. At expansion values, +3%, the obtained AHC in KMn_3_Sb_5_ and RbMn_3_Sb_5_ also exhibits a reduction in the values with σ_*xy*_ components of 145 and 229 S·cm^–1^, respectively. On the contrary, the CsMn_3_Sb_5_ crystals show an enhancement of the anomalous conductivity up to σ_*xy*_ = 442 S·cm^–1^ at +3%, but showing a tangible enhancement of σ_*xy*_ = 586 S·cm^–1^ at +2%. Thus, despite the quasi-linear displacements of the gapped points and the electronic structure as a function of strain, as shown in Figure S2, this unexpected behavior can originate in the complex relationship between the electronic density of states, included in the *f*_*n*_(**k**) term in Kubo’s relationship, and the Ω_*z*_ as a function of the strain that strongly depends on the values of energy gaps in the gapped crossings and the band repulsion in the presence of SOC and magnetically active sites. This leads to the displacement and modification of the curve profiles, as shown in Figure 5, of σ_*xy*_ as a function of the chemical potential extracted for different *xy*-plane strain values.

**Figure 5:**
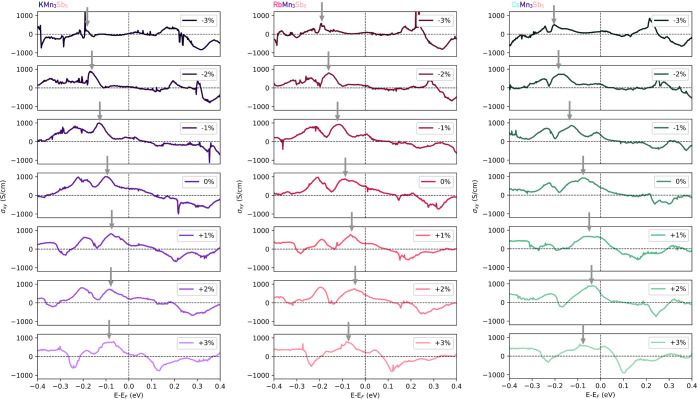
Estimated anomalous Hall conductivity, σ_*xy*_ component, computed as a function of the chemical potential near Fermi energy, here at *E*_*F*_ = 0.0 eV for notation. In this case, we considered the *z*-axis aligned ferromagnetic ordering for the KMn_3_Sb_5_, RbMn_3_Sb_5_, and CsMn_3_Sb_5_ compounds within the *P6/mm′m′* magnetic space group (MSG. 191.240). All of the calculations were obtained for strain values ranging from −3% to +3% strain values.

In Figure 6, we present the Ω_*z*_ Berry curvature component computed in CsMn_3_Sb_5_ for exemplification. The Ω_*z*_ curves in KMn_3_Sb_5_ and RbMn_3_Sb_5_ show similar trends and, therefore, are not shown in the document. Moreover, the Ω_*x*_ and Ω_*y*_ components are neglected as those are zero by symmetry considerations due to the time-reversal symmetry breaking that lies along the *z*-axis. At first glance, in the unstrained CsMn_3_Sb_5_ case, η = 0%, it can be observed that a strongly divergent Ω_*z*_ component is localized at the *K*- and *H*-high-symmetry points. These are in full agreement with the gapped Dirac points that lie at the Fermi level, as can be appreciated in Figure S3 in Supporting Information.^71^ This added to the split bands that also add to the Berry curvature integration. It is worth mentioning that such values extend up to approximately −6000 Å^2^, although the Berry curvature is displayed only up to −800 Å^2^ to make the visualization of smaller contributions that appear along other Brillouin zone paths. Finally, despite several Weyl points are identified in the vicinity of the *K*- and *H*-points, as observed in Figure S3b in Supporting Information,^71^ these are considerably far from the Fermi level, close to 140 meV and below −200 meV and therefore, do not present a tangible contribution to the Berry curvature shown in Figure 6.^79^ The latter is in contrast with the observed nature of the AHC, in which the intrinsic σ_*xy*_ measured is related to the Weyl points in the proximity of the Fermi level that, in turn, induces a nonzero large Berry curvature. In such a case, the nodal ring connecting positive and negative Weyl nodes is opened due to the SOC contribution and therefore, the gapped nodal lines, connected by the Weyl nodes are contributing considerably to the Berry curvature.^15^ In the case of the strained CsMn_3_Sb_5_, we noticed that at compression strain, η = −3%, the Ω_*z*_ is strongly reduced, explaining the considerable decrease in the AHC σ_*xy*_ component. Interestingly, the strong Ω_*z*_ peaks at *K* and *H* points disappear because of the closing of the gapped Dirac points at those points and the displacement in energy to lower values, below the Fermi, of such points, see Figure S3 in Supporting Information.^71^ At the expansion strain values, η = +3%, we observed several features associated with the contribution of the Berry curvature along the BZ path that are conserved in comparison to the unstrained crystals. Similarly, the peaks at *K* and *H* are reduced due to the displacement of these points in energy as a function of the applied epitaxial strain. Nevertheless, the reason for the slight enhancement of the σ_*xy*_ values needs further studies to better comprehend the entanglement between the electronic distribution and Berry curvature under strain in the Kagome compounds.

**Figure 6:**
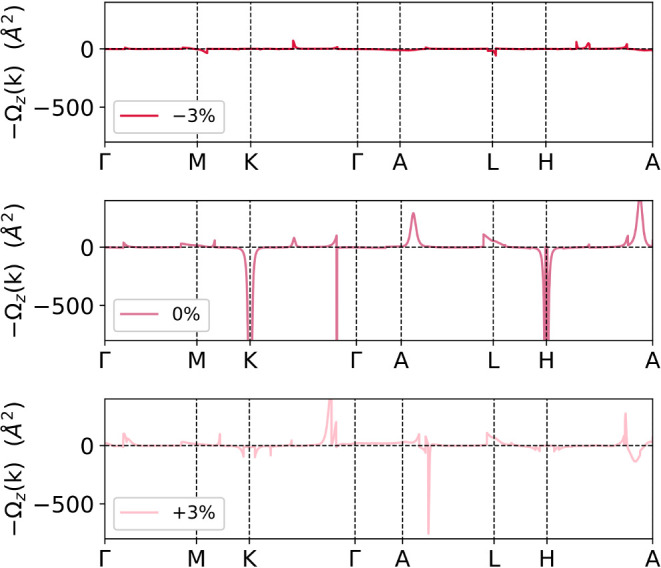
Computed Berry curvature *z*-component, Ω_*z*_, in the CsMn_3_Sb_5_ for the −3%, 0, and +3% strain values. In this case, we considered the *z*-axis aligned ferromagnetic ordering within the *P6/mm′m′* magnetic space group (MSG. 191.240).

Furthermore, we have also estimated the symmetry-allowed Ω_*z*_ Berry-curvature component projected band structure along the BZ path, see Figure 6S in the Supporting Information.^71^ The latter aims to facilitate the identification of sinks and sources of the tangible Ω_*z*_ component. As can be noted, for all of the *A*Mn_3_Sb_5_ compounds under the effect of the epitaxial strain, the largest Ω_*z*_ contributions, near the Fermi level, are located in the gapped nodal crossings along several points in the BZ path. The largest contributions in the *E*_*F*_ vicinity come from the *K* and *H* points, in good agreement with the discussion associated with Figure 6. Additional contributions can be observed in the *L*-point and other bands, for example, along the Γ–*A* path. The latter shows a large contribution due to the split bands induced by the SOC effect, added to the Zeeman splitting in the (001) ferromagnetic Kagome lattices. Finally, it can be appreciated that the shifting of the gapped points in *K* and *H* as a function of strain moving toward lower (higher) energy values for compression (expansion) strain, is in full agreement with the reduction of the Ω_*z*_ presented in Figure 5.

## Conclusions and General Remarks

We performed theoretically based first-principles density-functional calculations to delve into the *A*-site and *xy*-plane epitaxial strain effect on the structural, dynamical, electronic, and magnetic properties of the novel Mn-based Kagome metal *A*Mn_3_Sb_5_ (*A* = K, Rb, Cs) family. Our findings showed that the *A*Mn_3_Sb_5_ compounds are both structurally and vibrationally stable for *A* = K, Rb, and Cs, without and under expansion and compression strain. Despite the presence of in-plane magnetic frustration and the allowed presence of noncollinear antiferromagnetic orderings, the *c*-axis stacking resolves the frustration in an out-of-plane ferromagnetic ground state in the primitive cell. Nonetheless, as the **q** = (0,0,1/2) magnetic propagation vector is considered, the KMn_3_Sb_5_ and the RbMn_3_Sb_5_ display a weak tendency toward the *A*-type AFM ordering. In contrast, the CsMn_3_Sb_5_ member of the family shows a preference for a full ferromagnetic state. As expected from the magnetically active Kagome lattice, the electronic structure, in all of the compounds, exhibits multiple gapped nodal crossings near the Fermi level, in agreement with the massive Fermions phenomenon in magnetically active Kagome lattices. In terms of the epitaxial strain, there is no appreciable trend on the AHC, for example, in the KMn_3_Sb_5_ and RbMn_3_Sb_5_, either expansion or compression tends to decrease the σ_*xy*_ values, meanwhile it is increased on CsMn_3_Sb_5_ up to 442 S·cm^–1^ at +3% of expansion reaching a maximum of 586 S·cm^–1^ at +2%. The magnetic moment increases with expansion strain, suggesting an increase in electronic localization with the Mn–Mn distance. Then, in terms of the expected ferromagnetic ground state, the out-of-plane easy axis, and the anomalous Hall conductivity, σ_*xy*_, our results targeted the CsMn_3_Sb_5_ as the best ideal candidate among these magnetically active 2D Kagome materials studied. Finally, we truly hope that our results will motivate experimentalists and theoreticians alike to pursue further studies on this magnetically active *A*Mn_3_Sb_5_ family of Kagome compounds.

## Supplementary Material



## Data Availability

Data produced and used in the development of this work will be made available from the author upon reasonable request.

## Notes

The Supporting Information includes phonon dispersion curves for AMn_3_Sb_5_ (A = K, Rb, Cs) under epitaxial strain (±3%) as well as the electronic band structures for CsMn_3_Sb_5_ demonstrating the energy tuning of gapped Dirac points at *K* and *H* high-symmetry points under strain; spin-polarized band structures showing the s_*z*_ spin component for unstrained crystals; and the Ω_*z*_ Berry curvature component projected onto the band structures for all compounds, highlighting the contributions from gapped nodal crossings and SOC-induced splitting along the Γ–*A* path (PDF)
