# Supplementary material for: A‑Site and Epitaxial Strain Effect on the Properties of AMn3Sb5 (A = K, Cs, and Rb) Kagome Lattices
Source: ACS Omega. 2026 Jan 23;11(5):7988–97. doi: 10.1021/acsomega.5c10090 (PMC12903180; doi:10.1021/acsomega.5c10090)
Supplement: Supplementary file 1 [file ao5c10090_si_001.pdf]

Supplemental Material: A-site and epitaxial strain effect on the properties of $A\mathrm{Mn_3Sb_5}$ (A = K, Cs, and Rb) Kagome lattices

Andrés Camilo García Castro*,† and Wilfredo Ibarra Hernández*,‡

†School of Physics, Universidad Industrial de Santander, SAN-680002, Bucaramanga (Santander), Colombia.

‡Facultad de Ingeniería, Benemérita Universidad Autónoma de Puebla, Apartado Postal J-39, 72570, Puebla (Puebla) México.

E-mail: acgarcia@uis.edu.co; wilfredo.ibarra@correo.buap.mx

In Fig. S1 are displayed the phonon dispersion curves obtained in the $\mathrm{KMn_3Sb_5}$, $\mathrm{RbMn_3Sb_5}$, and $\mathrm{CsMn_3Sb_5}$, from top to bottom. In all the cases, the dispersions were computed at xy-strain values of -3% and +3%.

In Fig. S2, the electronic band structure obtained in the $\mathrm{CsMn_3Sb_5}$ Kagome compound under the effect of the epitaxial strain of the xy plane is presented. Here, the structure is shown for the -3%, 0%, and $+3\%$ strain values. As observed, there is a tuning of several electronic states going to lower energies when going from -3% to $+3\%$. However, the gapped Dirac points, at K- and H-high-symmetry points, are located below, for -3%, and above, for $+3\%$, demonstrating the feasible tuning and modification of the topological points.

In Fig. S3, the s_z spin component is presented in the spin-polarized band structure computed in the $A\mathrm{Mn_3Sb_5}$ unstrained Kagome crystals. The s_x and s_y are not shown because the spin moments are entirely aligned along the z-axis and therefore, there is no tangible

contribution of these components in the spin texture.

Ω_z Berry curvature component, projected in the band-structure, is presented in Fig. S4 for all the $A\mathrm{Mn_3Sb_5}$ compounds under the effect of the epitaxial strain. In this case, it can be noted that the largest Ω_z, near the Fermi level, are located in the gapped nodal crossings. Other bands, for example, along the Γ–A path, show a tangible contribution due to the splitted bands due to the SOC effect added to the Zeeman splitting in the 001 Ferromagnetic Kagome lattices.

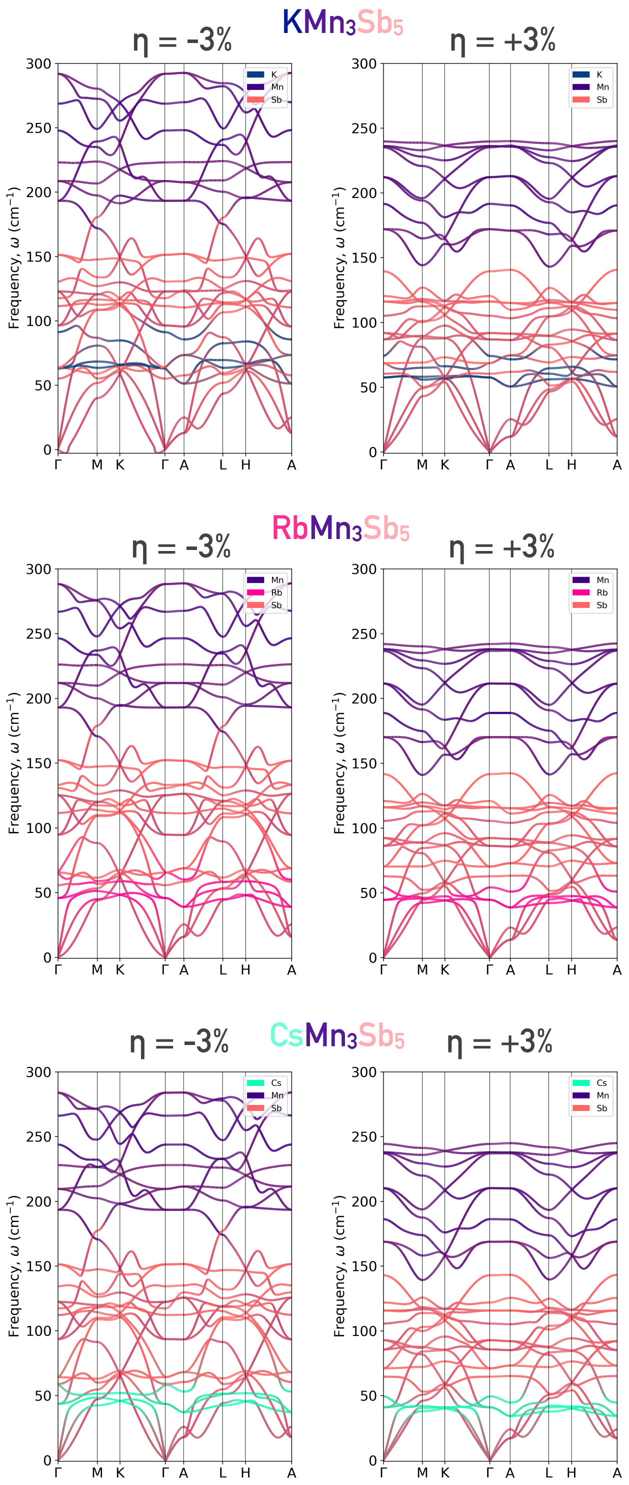

Figure S1: (Color online) Phononic band structure computed in the $A\mathrm{Mn_3Sb_5}$ (A = K, Rb, and Cs) Kagome compounds under the effect of the xy-plane epitaxial strain. Here, the phonon dispersion is displayed along the typical high-symmetry path for the $P6/mmm$ (SG. 191) hexagonal space group. In this case, the -3% and +3% strain values are considered.

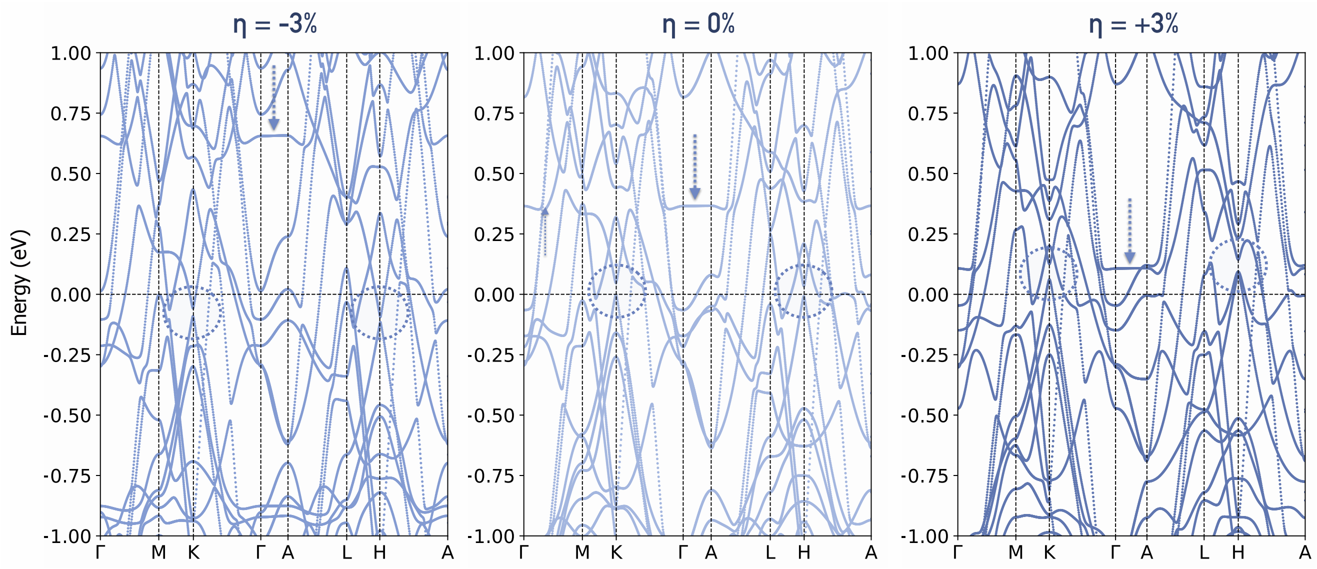

Figure S2: (Color online) Electronic band structure computed in the $CsMn_3Sb_5$ Kagome compound under the effect of the xy-plane epitaxial strain. Here, the electronic structure is plotted along the typical high-symmetry path for the $P6/mmm$ (SG. 191) hexagonal space group. In this case, the -3%, 0%, and $+3\%$ strain values are considered.

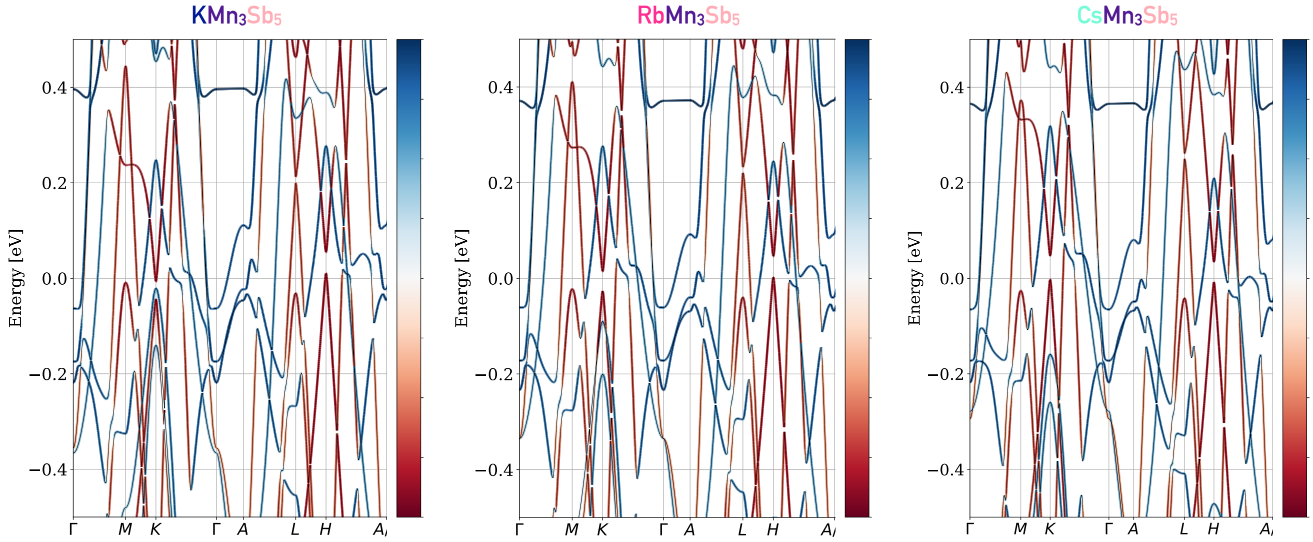

Figure S3: (Color online) s_z spin-projected electronic band structure computed in the unstrained KMn_3Sb_5, $RbMn_3Sb_5$, and $CsMn_3Sb_5$ Kagome compounds. Here, the up and down spin polarizations are denoted in blue and red colors, respectively.

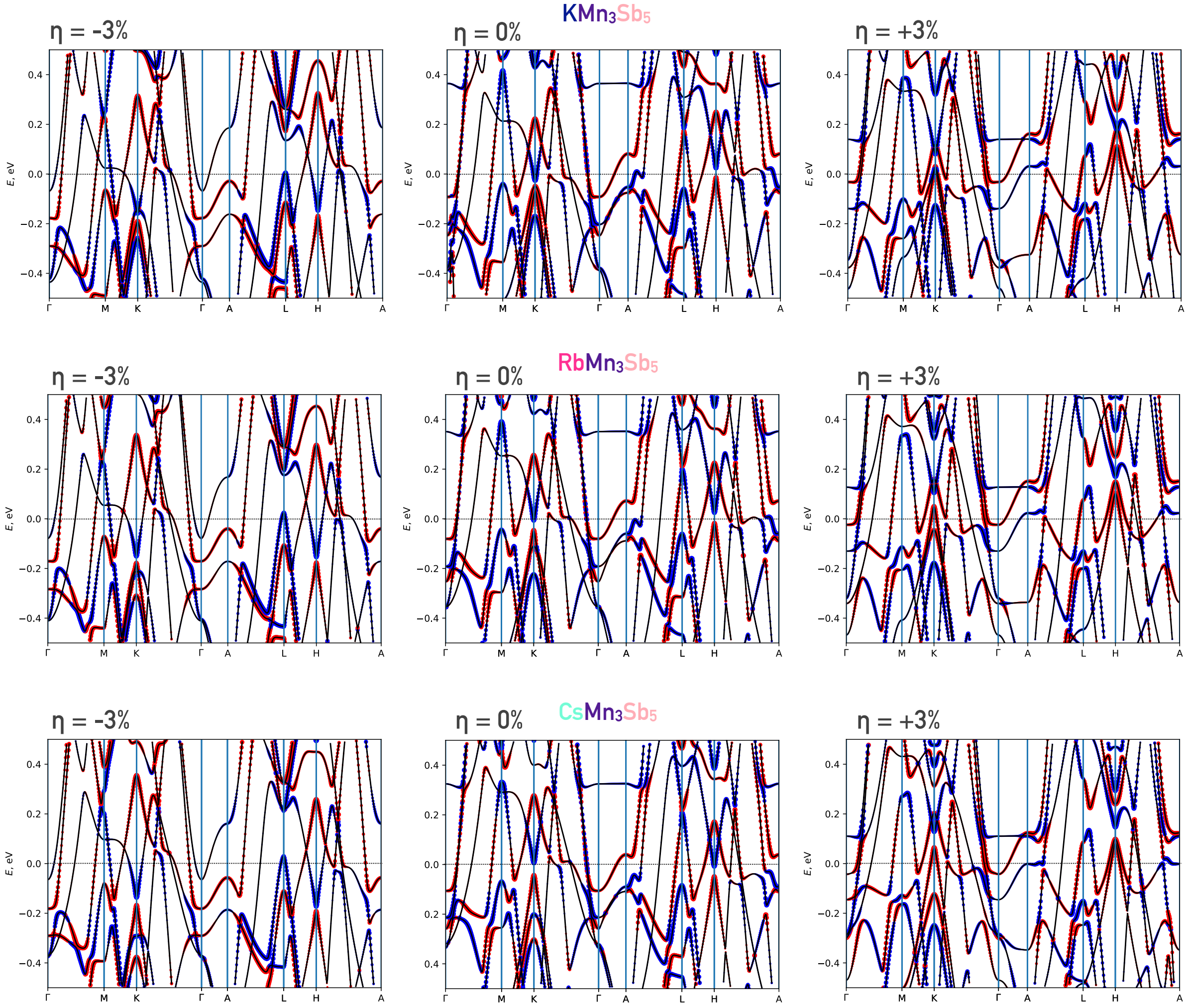

Figure S4: (Color online) Berry-curvature z-component (Ω_z)projected onto the band-structure computed in the AMn$_3$Sb$_5$ (A = K, Rb, and Cs) Kagome compounds, considering the xy-plane epitaxial strain. Here, the red and blue colors represent the positive and negative values of Ω_z values, respectively.
